# Laparoscopic versus laparotomic surgical treatment in apparent stage I ovarian cancer: a multi-center retrospective cohort study

**DOI:** 10.1186/s12957-024-03345-1

**Published:** 2024-02-22

**Authors:** Jing Zhang, Meiyan Li, Lan Feng, Yinjun Zhai, Lin Wang, Yuancao Chen

**Affiliations:** 1 Department of Gynecology, The First Affiliated Hospital of Xingtai Medical College (Xingtai First Hospital), No. 376 Shunde Road, Xiangdu District, Xingtai, Hebei Province 054000 People’s Republic of China; 2 Department of Gynecology, Handan Central Hospital, Handan, Hebei Province People’s Republic of China; 3 Department of Intervention, The First Affiliated Hospital of Xingtai Medical College (Xingtai First Hospital), Xingtai, Hebei Province People’s Republic of China; 4 Department of Gastrointestinal Surgery, The First Affiliated Hospital of Xingtai Medical College (Xingtai First Hospital), Xingtai, Hebei Province People’s Republic of China

**Keywords:** Early-stage ovarian cancer, Laparoscopic surgery, Laparotomic surgery, Perioperative complication, Survival outcomes

## Abstract

**Background:**

Laparoscopic treatment shows non-inferior survival outcomes and better surgical outcomes in apparent stage I ovarian cancer (OC) in some studies but has not been well defined.

**Methods:**

We conducted a retrospective study of patients with apparent stage I OC treated in two hospitals between 2012 and 2022. The surgical and oncologic outcomes were evaluated between patients receiving laparoscopic and laparotomic surgery.

**Results:**

We identified 37 patients with apparent stage I OC, including 15 (40.5%) serous carcinomas, 9 (24.3%) mucinous cancers, 3 (8.1%) endometroid cancers, 2 clear cell carcinomas, and 8 (21.6%) non-epithelial cancers. Sixteen patients received laparoscopic surgery and the other 21 patients underwent laparotomic surgery. The median age (44.5 vs. 49.0 years), mean mass size (10.5 vs. 11.3 cm), and median follow-up time (43.5 vs. 75.0 months) showed no statistically significant differences between patients in laparoscopic and laparotomic groups (all *P* > 0.05). All the patients underwent comprehensive surgical staging surgery, and the mean surgical time (213.5 vs. 203.3 min, *P* = 0.507), number of lymph nodes sampling (18.6 vs. 17.5, *P* = 0.359), proportion of upstaging (12.5% vs. 19.0%, *P* = 0.680), and postoperative complications (no Accordion Severity Grading System grade ≥ 3) were comparable between two surgical groups. Moreover, patients in the laparoscopic group had significantly less intraoperative blood loss (231.3 vs. 352.4 mL, *P* = 0.018), shorter interval between surgery and postoperative adjuvant chemotherapy (7.4 vs. 9.5 days, *P* = 0.004), shorter length of hospital stay (9.9 vs. 13.8 days, *P* < 0.001) than those treated with laparotomic surgery. During a median follow-up of 54.0 months, 9 (24.3%) relapsed and 1 (2.7%) died, with a 5-year recurrence-free survival (RFS) and disease-specific survival (DSS) rate of 70.6% and 100%, respectively. However, the 5-year RFS (93.3% vs. 58.8%, *P* = 0.084) and DSS (100% vs. 100%, *P* = 0.637) rates did not significantly differ between the two groups.

**Conclusion:**

Laparoscopic surgical treatment had less intraoperative blood loss, earlier postoperative adjuvant chemotherapy administration, shorter hospitalization time, and non-inferior survival outcomes in apparent stage I OC when compared with laparotomic surgery.

## Introduction

Ovarian cancer (OC) is the second leading cause of cancer mortality in gynecologic malignancies worldwide, accounting for approximately 5% of all cancer-related deaths among women [1]. Approximately 70% of the OC were advanced stage upon diagnosis and the 5-year overall survival rate was about 40–50% [2, 3]. In China, there were more than 50 thousand newly diagnosed OC patients and 26 thousand OC-related deaths in 2019 [4]. Moreover, both the incidence and mortality rate of OC show an increasing trend in the past 15 years [5]. The standard surgical approach for advanced OC is midline laparotomy, however, whether laparoscopic surgery could be an alternative option for early-stage OC remains controversial [3]. Determining the role of minimally invasive surgery in early-stage OC will help to optimize clinical management because about 20% of OC can be classified as FIGO stage I [6].

There have been several studies investigating the safety of laparoscopic surgery in early-stage OC [7–15]. Most of these studies demonstrated a comparable survival outcome between patients receiving laparoscopic and laparotomic surgery, and patients who underwent laparoscopic surgery had less blood loss, shorter length of hospitalization, and earlier postoperative recovery [8–15]. However, there were different inclusion criteria in selecting early-stage OC patients [8–11] and some inconsistent results on surgical outcomes [8, 10, 15], and the follow-up time in some studies was within two years [9–11]. Moreover, none was published within the past five years and only one was reported from China but published nearly 10 years ago [13, 14]. Two systematic reviews also demonstrated that currently there was no prospective study regarding the feasibility of laparoscopic staging surgery in stage I OC [12, 16]. Data on the Chinese population concerning this issue remains insufficient, and research on laparoscopic surgical treatment for apparent stage I ovarian cancer patients still needs to be further evaluated and updated.

Therefore, to evaluate the safety of laparoscopic surgery for apparent stage I ovarian cancer patients in China, we conducted a multi-center retrospective study. The surgical and oncologic outcomes were evaluated between patients receiving laparoscopic and laparotomic surgery.

## Materials and methods

The Ethics Committee of the Xingtai First Hospital and Handan Central Hospital approved this study. Patients with early-stage ovarian cancer treated in these two hospitals from January 1st, 2012 to December 1st, 2022 were included in this study. OC patients treated in our two hospitals were screened and only those with apparent stage I diseases before surgery as well as complete medical data were enrolled. Moreover, patients who had a follow-up time of less than 12 months were excluded. Data including the demographics, clinical and pathological characteristics, surgical treatments, and survival outcomes were extracted from eligible patients. We divided patients into two groups, the laparoscopic group and the laparotomic group, based on their surgical options. The baseline characteristics, surgical, and oncologic outcomes were compared between the two subgroups. In this study, the baseline characteristics included patients’ age at diagnosis (years), body mass index (BMI, kg/m^2^), mass sizes (cm), preoperative CA125 levels, FIGO stage, and pathologic subtypes. The surgical outcomes included surgical time (min), intraoperative blood loss (mL), number of lymph nodes sampling, proportion of upstage after surgical staging and postoperative complications (grading according to the Accordion Severity Grading System [ASGS]), interval between surgery and adjuvant chemotherapy, length of hospital stay. Besides, recurrence-free survival (RFS) and disease-specific survival (DSS) were the oncologic outcomes. RFS was defined as the date from initial treatment intervention to confirmed tumor relapse and DSS was defined as the time from the date of the initial treatment to death related to the tumor or final follow-up. Subgroup analysis was performed based on the tumor pathologic subtypes.

### Statistical analysis

Variables were described according to their distributions, namely, means ± standard deviation (range) or as medians and interquartile ranges (IQRs) for continuous variables, and counts (percentages) for discrete variables. An Independent-sample T-test was used to identify differences between the two subgroups, while a chi-squared test or Fisher’s exact test was used to compare categorical variables. The survival analyses were performed by using the Kaplan–Meier method (log-rank test). We set the statistically significant cut-off as a *P* value < 0.05 (two-tailed). Statistical analyses were conducted using SPSS (version 21.0; SPSS Inc., Chicago, IL, USA) or GraphPad Prism (version 8.0) software.

## Results

A total of 37 patients met the inclusion criteria after screening. Asymptomatic pelvic mass was identified by imaging examination (22/37, 59.5%), followed by mild pelvic discomfort (13/37, 35.1%), and two patients manifested acute abdominal pain caused by tumor rupture. The median age at diagnosis among them was 48.0 years (range: 14 -79), and the median mass size was 9.1 cm (range: 1.9 – 25.0). The demographic and clinical characteristics are listed in Table 1.

**Table 1:** The demographic and clinical characteristics between the overall cohort and two comparison groups

	Laparoscopic (*N* = 16)	Laparotomic (*N* = 21)	*P*—value	Overall (*N* = 37)
Age (y)	44.5 ± 15.3/44.5 (14–73)	48.33 ± 15.6/49.0 (16–79)	0.460	46.7 ± 15.4/48.0 (14–79)
BMI (kg/m^2^)	24.6 ± 4.5/23.4 (15.6 – 35.2)	23.8 ± 2.9/24.0 (17.7 – 29.0)	0.524	24.1 ± 3.6/23.4 (15.6 – 35.2)
Mass size (cm)	10.5 ± 5.9/9.0 (2.3 – 25.0)	11.3 ± 5.6/10.5 (1.9–21.5)	0.690	11.0 ± 5.6/9.1 (1.9 – 25.0)
Pre-CA125 level (mean/median, U/ml)	782.3/39.1 (10.6 – 6735.6)	148.7/45.6 (9.4 – 1214.9)	0.137	422.7/45.6 (9.4 – 6735.6)
FIGO stage				
IA	1	2		3 (8.1%)
IC	13	15		28 (75.7%)
IIA	1	1		2 (5.4%)
IIB	1	3		4 (10.8%)
Pathology				
Serous	6	9		15 (40.5%)
Endometroid	1	2		3 (8.1%)
Mucinous	4	5		9 (24.3%)
Clear cell	1	1		2 (5.4%)
MGCT	0	3		3 (8.1%)
DG	-	1		1
YST	-	1		1
EC	-	1		1
AGCT	3	1		4 (10.8%)
ESS	1	0		1 (2.7%)
Follow-up time (m)	48.4/43.5 (14—100)	66.6/75.0 (17—117)	0.063	58.7/54.0 (14 – 117)

The age, follow-up time, and mass size were presented as mean ± standard deviation/median (range)

*Abbreviations*: *MGCT* malignant germ cell tumors, *AGCT* adult granulosa cell tumors, *ESS* endometrial stromal sarcoma

Further auxiliary examinations revealed no apparent extra-ovarian metastases among these 37 patients. After the suspected diagnosis of ovarian tumor, patients were treated by surgical exploration, of whom 16 received laparoscopic surgery and the other 21 underwent laparotomic surgery. Postoperative pathology revealed that serous carcinoma (15 cases, 40.5%) was the most common pathology subtype, followed by mucinous carcinoma (9 cases, 24.3%), non-epithelial cancer (8 cases, 21.6%), endometroid cancer (3 cases, 8.1%), and clear cell carcinoma (2 cases, 5.4%). Among the 8 patients diagnosed with non-epithelial cancer, there were 4 adult granulosa cell tumors (AGCT), 3 malignant germ cell tumors (MGCT, one each for dysgerminoma, yolk sac tumor, and embryonic carcinoma), and 1 low-grade endometrial stromal sarcoma (LGESS), respectively. Moreover, there were 19 high-grade carcinomas within these 37 patients. However, no statistically significance difference in histological subtype (4 non-epithelial OC patients in each group, *P* = 0.705) and high-grade tumor proportion (8 patients vs. 11 patients, *P* = 1.000) between the laparoscopic and laparotomic groups.

The mean surgical time (213.5 vs. 203.3 min, *P* = 0.507), number of lymph nodes sampling (18.6 vs. 17.5, *P* = 0.359), and proportion of upstaging (12.5% vs. 19.0%, *P* = 0.680) were comparable between two surgical groups. Fertility-sparing surgery was performed in 3 patients (2 adult granulosa cell tumor and 1 ovarian yolk sac tumor, of which one granulosa cell tumor patient in laparoscopic group and the other two patients in laparotomic group). Tumor stage in two patients had been upstaged in the laparoscopic group, of which one was upstaged from stage I to IIA after surgical staging due to tumor involvement of the ipsilateral ovary and fallopian tube, and the other one because of micrometastasis identified by biopsy of pelvic peritoneum. Similarly, four patients receiving laparotomic surgery were upstaged from stage I to stage II, of which one was due to tumor infiltrating ipsilateral salpinx and three had tumors involving the pelvic peritoneum. It should be noted that there was each one tumor rupture during the surgery in two groups, without statistically significant difference (6.3% vs. 4.8%, *P* = 1.000). For the other 26 patients who were staged as IC diseases, 11 had preoperative tumor rupture and the rest 15 patients had tumor cells found in ascites/peritoneal washings. Moreover, there was no significant difference in the incidence of postoperative complications. Notably, no ASGS grade 3 or higher postoperative complications were noted between the two groups. However, patients in the laparoscopic group had significantly less intraoperative blood loss (231.3 vs. 352.4 mL, *P* = 0.018), shorter interval between surgery and postoperative adjuvant chemotherapy (7.4 vs. 9.5 days, *P* = 0.004), shorter length of hospital stay (9.9 vs. 13.8 days, *P* < 0.001) than those treated with laparotomic surgery (Table 2). Platinum-based chemotherapy was applied in all but one AGCT and one LGESS patient. Three MGCT patients received bleomycin, etoposide, and cisplatin chemotherapy, and other 2 AGCT patients received paclitaxel plus carboplatin chemotherapy. For the 29 epithelial OC patients, paclitaxel plus carboplatin was given in 25 patients, and four mucinous cancer patients received oxaliplatin plus capecitabine.

**Table 2:** The surgical details, adjuvant chemotherapy, and outcomes between laparoscopic and laparotomic groups

	Laparoscopic (*N* = 16)	Laparotomic (*N* = 21)	*P*—value
Surgical time (min)	213.5 ± 37.4	203.3 ± 51.0	0.507
Intraoperative blood loss (ml)*	231.3 ± 132.5	352.4 ± 156.9	0.018
Intraoperative transfusion	3 (18.8%)	3 (14.3%)	1.000
Number of LN sampling	18.6 ± 2.8 (*N* = 11)	17.5 ± 3.0 (*N* = 18)	0.359
Pelvic LN	12.6 ± 4.0	11.9 ± 3.5	0.676
Para-aortic LN	6.0 ± 1.8	5.6 ± 1.7	0.512
Intraoperative tumor rupture	1 (6.3%)	1 (4.8%)	1.000
Upstage	2 (12.5%)	4 (19.0%)	0.680
Postoperative complications			
ASGS grade 2	16 (100%)	21 (100%)	NS
ASGS grade 3 or higher	-	-	
Time to chemothearpy (d)*	7.4 ± 1.5	9.5 ± 2.7	0.004
Length of hospital stay (d)*	9.9 ± 1.6	13.8 ± 4.4	< 0.001

*Abbreviations*: *LN* lymph nodes, *NS* not significant, *ASGS* Accordion Severity Grading System

^*^P < 0.05

**Fig. 1:**
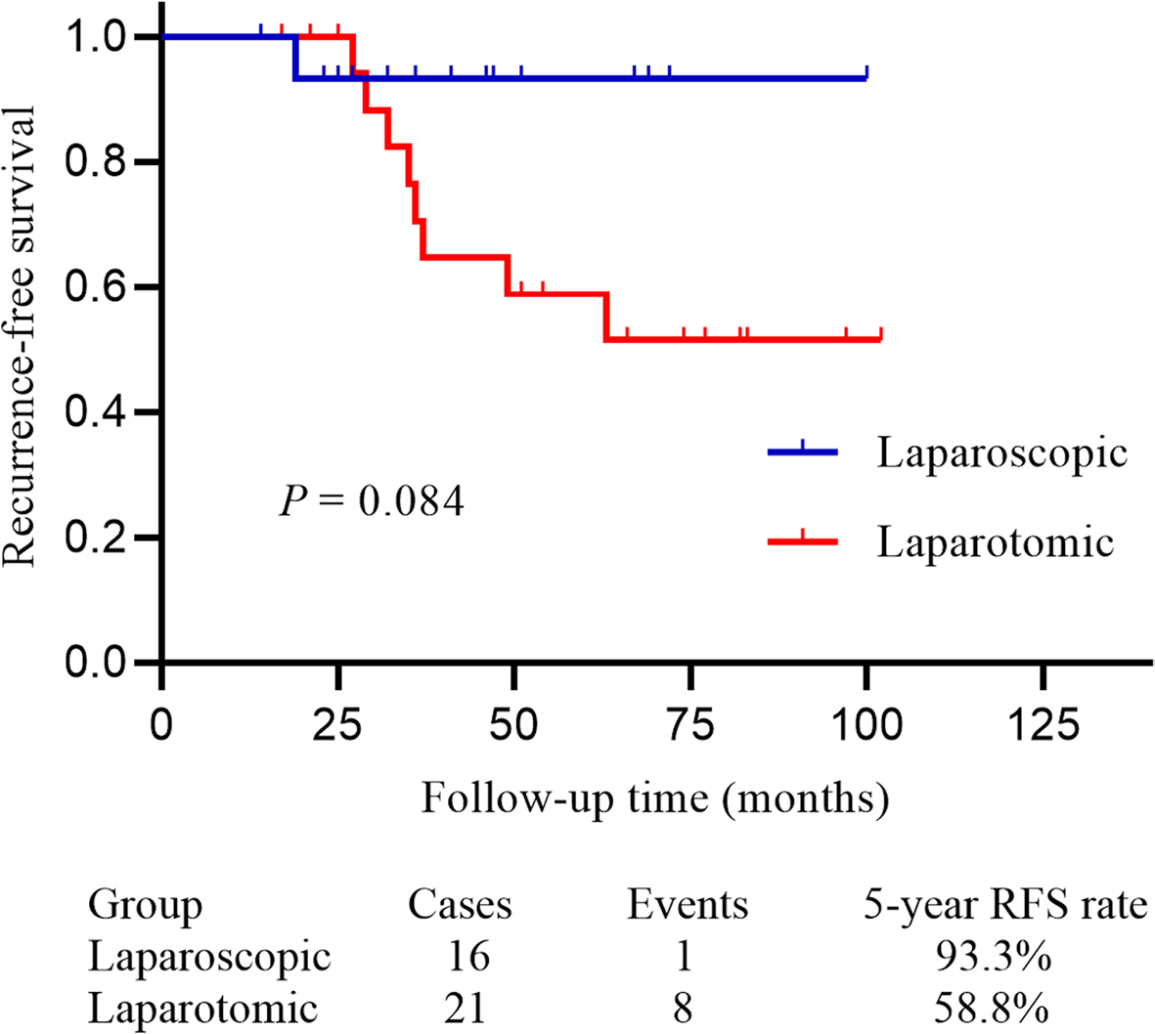
The RFS in apparent stage I OC patients treated with laparoscopic and laparotomic surgery showed by Kaplan–Meier curve

During the follow-up, 9 patients experienced relapses, of whom 1 was in the laparoscopic group and 8 in the laparotomic group, with a 5-year RFS rate of 70.6%. The 5-year RFS rates in the laparoscopic and laparotomic groups were 93.3% and 58.8%, respectively (*P* = 0.084, Fig. 1). Of the 8 relapsed patients in the laparotomic group, five were FIGO stage IC high-grade serous carcinoma, two were mucinous carcinoma (one stage IC and one stage IIB), and the rest one was FIGO stage IC AGCT initially treated with surgery only. Moreover, the only patient who experienced recurrence in the laparoscopic group was FIGO stage IC poor-differentiated clear cell carcinoma. Among them, two patients received repeat cytoreductive surgery followed by platinum-based chemotherapy after recurrence, and the other 7 patients were treated with platinum-based chemotherapy. Six patients achieved no evidence of disease (NED) after the first recurrence, of whom two experienced at least a second relapse. Of the other three patients, two had partial remission diseases and one had a progression disease and then succumbed to cancer after the first relapse. Second-line or posterior-line chemotherapy was subsequently given for these relapsed patients.

**Fig. 2:**
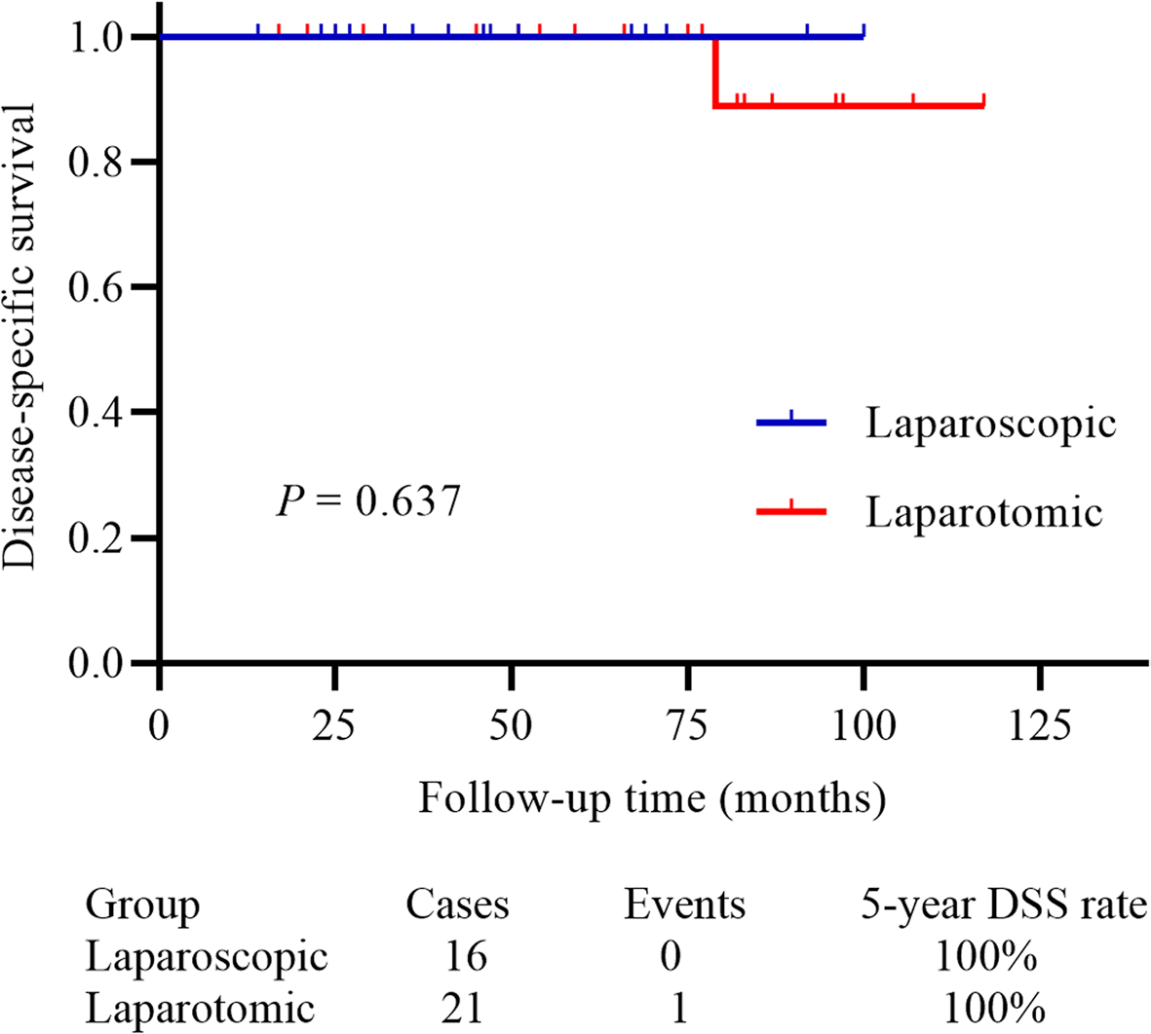
The DSS was comparable between patients in laparoscopic and laparotomic group

**Fig. 3:**
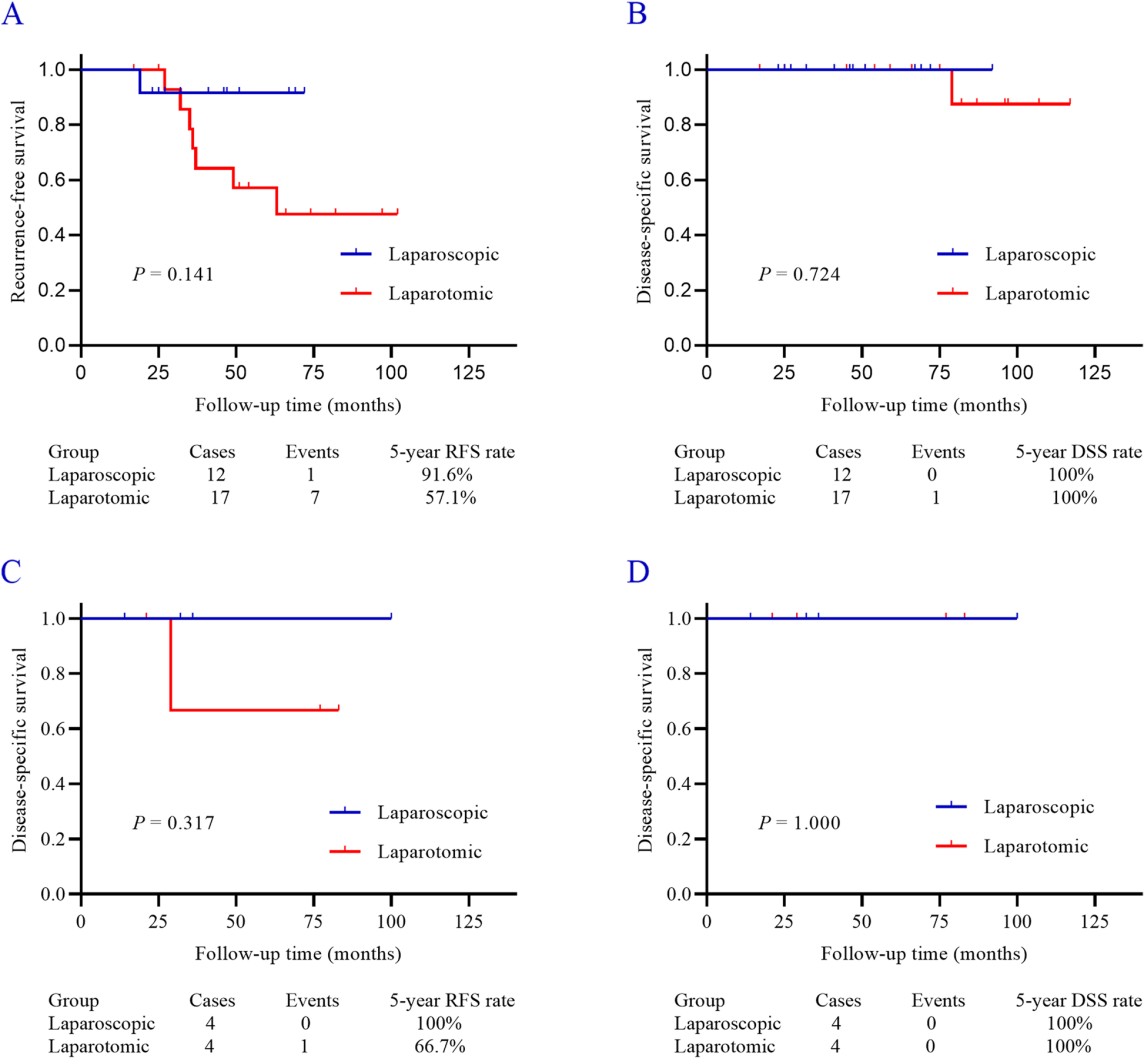
The survival outcomes in subgroup analysis of this cohort. **A**-**B** The RFS and the DSS were comparable in apparent stage I epithelial OC underwent laparoscopic or laparotomic surgery. **C**-**D** Similar results were also noted in apparent stage I non-epithelial OC patients received laparoscopic or laparotomic surgery

At the end of the follow-up, only one patient died of the disease 79 months after diagnosis (in the laparotomic group), and the 5-year DSS rate was 100% after a median follow-up of 54.0 months. The median follow-up time was 43.5 and 75.0 months (*P* = 0.063) in the laparoscopic and laparotomic groups, respectively. There was no statistically significant difference between patients receiving laparoscopic and laparotomic surgery (*P* = 0.637, Fig. 2). Subgroup analysis according to the tumor histological subtypes was subsequently performed. In 29 epithelial OC patients, 12 and 17 of them received laparoscopic and laparotomic surgery, respectively. The Kaplan–Meier analysis also showed that no statistical difference in both RFS (*P* = 0.141, Fig. 3A) and DSS (*P* = 0.724, Fig. 3B). Similarly, in other 8 non-epithelial OC patients’ subgroup, both the RFS (*P* = 0.317, Fig. 3C) and DSS (*P* = 1.000, Fig. 3D) were comparable between two surgical options.

## Discussion

Our study originally from two large regional medical centers revealed that laparoscopic surgical treatment for apparent stage I OC had less intraoperative blood loss, shorter interval between surgery and adjuvant chemotherapy, and shorter hospitalization times when compared with those receiving laparotomic surgery. The 5-year RFS and DSS rates were comparable between the two groups, without statistically significant differences. Therefore, laparoscopic surgery could be an alternative option in the management of apparent stage I OC.

Previously, several studies have evaluated the role of minimally invasive surgery in early-stage OC patients since the rapid development of laparoscopic technology [8, 14, 15]. In 2004, three studies [17–19] demonstrated the technical safety and efficacy of laparoscopic management of early-stage OC, but they did not have laparotomic control groups. More recently, Gallotta et al. showed that only grade 3 tumor was the negative prognostic factor in 254 early-stage EOC patients with a median follow-up of 61 months, rather than the surgical option [20]. They also stated that no statistical differences in survival outcomes between patients underwent robotic surgery and conventional laparoscopic surgery in early-stage OC [21]. Chi et al. [7] in 2005 first reported a comparative study focusing on the surgical and oncologic outcomes between early-stage OC patients treated with laparoscopic or laparotomic surgery. Most studies showed that early-stage OC patients receiving laparoscopic surgery had shorter hospitalization times [7, 8, 13], less blood loss [7, 10, 11], and shorter intervals to postoperative adjuvant chemotherapy [10, 11, 13], although these differences in some studies were not statistically significant [9]. Moreover, researchers observed that minor postoperative complications in patients underwent laparoscopic surgery than laparotomic surgery [8, 10–12]. However, inconsistent result was noted in the operation time between the two surgical options, some research revealed that operation duration was significantly longer in the laparoscopic group than laparotomic group [7, 8, 12], while others showed a converse finding [10], and some found no significant difference the rest studies [13]. Results from our study were consistent with most of the previous research that patients had significantly shorter hospitalization times, less blood loss, and comparable operative duration. The inconsistency in some surgical outcomes, especially for the surgical duration time, could be explained by the intra-hospital differences in laparoscopic experiences and the rapid development of minimally invasive surgery in gynecologic malignancies since most recent studies showed shorter or no difference in operation time between two groups [10, 13, 15]. Nonetheless, the comparable surgical outcomes that originated from this series of cohort studies emphasized the surgical safety and advantages in laparoscopic management for early-stage OC.

Although the current evidence shows that laparoscopic surgery for early-stage OC is technically feasible, the main question is whether it could guarantee non-inferior oncologic outcomes when compared with laparotomic surgery, the standard surgical approach for ovarian malignancies [3]. There are three major concerns on the laparoscopic treatment for apparent stage I OC: (1) tumor progression due to CO_2_ during pneumoperitoneum [22]; (2) risk of trocar sites metastases [23]; (3) increased risk of tumor rupture/upstaging that leading to decreased oncologic outcomes [7]. However, the effects of tumor progression induced by pneumoperitoneum originated from cells or animal models [22, 24]. However, data from laparoscopic treatment for non-metastatic colorectal cancer showed significantly better both survival and surgical outcomes than open surgery [25]. These inconsistent results remind the proper interpretation of pneumoperitoneum on survival outcomes, and it should not be arbitrarily regarded as an independent risk factor. Besides, Zivanovic et al. [26] in 2008 reported that only 1.96% (15 of 797) adnexal/peritoneal malignancies underwent laparoscopic surgery and developed trocar sites metastases, and all these patients coexisted with metastatic lesions (FIGO stage IIIC) during the surgery. Subsequent cohort studies restricted to early-stage OC patients who underwent laparoscopic surgery showed no port-site metastases [12–15]. Our study also found none experienced trocar-site recurrence, which again confirmed the oncologic safety of this issue.

The incidence of tumor rupture or upstaging due to laparoscopic surgery has also been investigated, with an estimated intraoperative tumor rupture rate of approximately 20% [27] and upstaging rate of 6%—43% [12, 14]. Bogani et al. in 2014 [12] reported the rate of spillage in laparoscopic and open groups was similar (6/35 vs. 4/32, *P* = 0.59). They subsequently performed a meta-analysis that enrolled 6 comparative research of 152 laparoscopic and 241 open staging early-stage OC patients. Neither the spillage (OR 0.78, 95% CI 0.35 – 1.73) nor the upstaging risk (OR 0.7, 95% CI 0.38 – 1.27) was significantly different between patients receiving two surgical options [12]. Recently, a large propensity-matched cohort study also found that there was no statistically different upstaging rate in the laparoscopic or open group (20% vs. 26%, *P* = 0.63) [14]. Our research showed a comparable result that no significant difference in the tumor rupture (6.3% vs. 4.8%, *P* = 1.00) or upstaging rate (12.5% vs. 19.0%,* P* = 0.68) between apparent stage I OC patients receiving laparoscopic or laparotomic treatment. Therefore, laparoscopic in this specific OC sub-population is at higher risk of tumor rupture/upstaging may be a biased, intrinsic impression.

Our cohort demonstrated that both the 5-year RFS rate and the 5-year DSS rate were comparable between patients receiving laparoscopic and laparotomic surgery. The reason why patients in laparotomic groups seemed at higher risk of relapse may be attributed to the delay of postoperative adjuvant chemotherapy and the aggressive histologic subtypes [3]. Indeed, laparotomic patients had significantly longer intervals between surgery and adjuvant chemotherapy, and most relapsed patients had high-grade carcinoma or mucinous carcinoma in our study. This result was consistent with previous research that laparoscopic surgery showed non-inferior survival outcomes when compared with laparotomic treatment [7–15]. A systematic review summarized studies published till 2014 showed that the risk of recurrence in patients who underwent laparoscopic or open surgery was similar (OR 0.5, 95% CI 0.21 – 1.21) [12]. Although the median follow-up time for most of these studies was shorter than 3 years, several studies presented survival outcomes with more than 4 years of follow-up [12, 14]. Ditto et al. [14] reported that the 5-year RFS and DSS rates were no different in two surgical groups after a median follow-up of nearly 5 years in a cohort of 100 patients. The median follow-up time in our cohort was also relatively longer than other cohorts but with similar oncologic outcomes in two surgical options, and both the epithelial subgroup and non-epithelial subgroup showed consistent results. These findings suggested laparoscopic treatment indeed could be an alternative option in properly selected apparent stage I OC patients.

Nevertheless, it could be cautiously performed but several key points remain must be stated. Firstly, it should be emphasized that properly selecting apparent stage I patients for laparoscopic surgery is the cornerstone to guarantee promising survival outcomes. Moreover, laparoscopic surgery for these patients should be performed by experienced gynecologic oncologists and comprehensive preoperative evaluations must be conducted to minimize the possibility of metastatic diseases. Furthermore, a specimen bag should be routinely used in laparoscopic surgery to intactly remove the tumor, and laparotomy may be preferred in case of a tumor size larger than the specimen bag.

The multi-center study strengthened the reliability and feasibility of improving the clinical practice in managing patients with apparent stage I ovarian cancer. The retrospective nature of this study and the relatively small sample size were the two main limitations. Future prospective studies concerning this issue will help improve clinical management in patients with apparent stage I ovarian cancer.

## Conclusion

Laparoscopic surgical treatment for apparent stage I OC had less intraoperative blood loss, shorter intervals between surgery and adjuvant chemotherapy, and shorter hospitalization times when compared with those receiving laparotomic surgery. The survival outcomes were comparable among apparent stage I OC patients who underwent laparoscopic or laparotomic surgery.

## Data Availability

All data generated or analyzed during this study are included in this published article and supplementary files. The datasets used and/or analyzed during the current study can be obtained from the corresponding author upon reasonable request.

## Abbreviations

OC: Ovarian cancer
FIGO: International Federation of Gynecology and Obstetrics
RFS: Recurrence-free survival
DSS: Disease-specific survival
USO: Unilateral salpingo-oophorectomy
BSO: Bilateral salpingo-oophorectomy
H/BSO: Hysterectomy with bilateral salpingo-oophorectomy
